# Classification of myofibers using statistics of the helix angle: a novel approach to characterize the structure of the human heart

**DOI:** 10.1186/1532-429X-13-S1-O44

**Published:** 2011-02-02

**Authors:** Choukri Mekkaoui, Shuning Huang, Guangping Dai, Timothy G Reese, Aravinda Thiagalingham, Udo Hoffmann, Marcel P Jackowski, David E Sosnovik

**Affiliations:** 1 Harvard Medical School, Charlestown, MA, USA; 2 University of São Paulo, Institute of Mathematics and Statistics, São Paulo, Brazil

## Purpose

To characterize the microstructure of human/mammalian hearts using a statistical definition of fiber helix angle in the myocardial continuum.

## Introduction

The integration of primary eigenvectors in a diffusion field yields continuous tracts, to which it is useful to assign a single helix angle. However, the optimal approach to derive the helix angle of a continuous tract in the myocardium remains unclear. Strategies used in the brain and in prior tractographic studies in the heart (*Circ Cardiovasc Imaging.* 2009; 2(3): 206-12) may not be optimal for the myocardial continuum.

## Methods

Excised human, sheep and rat hearts (n=12) were studied. Diffusion tensor MRI (DT-MRI) of the human and sheep hearts was performed at 3T using 6 gradient-encoding directions; a b-value of 2000s/mm^2^; voxel-size=2x2x2mm^3^; TR/TE=8430/96ms; and 24 averages. DT-MRI of the rat hearts was performed at 4.7T using a b-value of 2000 s/mm^2^ and spatial resolution of 0.4x0.4x0.4 mm^3^. Fiber tracking was performed with a fourth-order Runge-Kutta approach. The helix angle assigned to each tract was defined in three ways: 1) Each voxel in the tract had its own (original) helix angle defined by its primary eigenvector, 2) the entire tract was classified by its median helix angle, or 3) by its mean helix angle.

## Results

**Figure 1:**
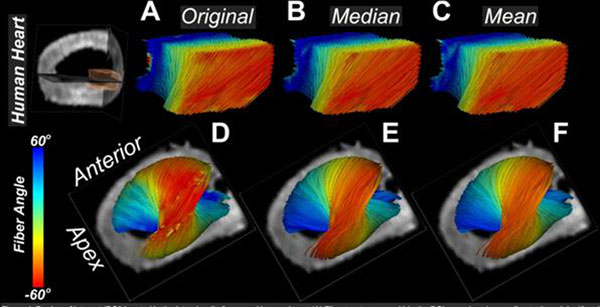
Region of interest (ROI) located in the lateral wall of a normal human heart. (A) Fiber tractograms within the ROI are colored according to the original/local helix angle. (B,C) Fiber tractograms colored according to (B) the median helix angle and (C) the mean helix angle computed along individual fiber trajectories. (D-F) Lateral view of myofibers passing through the ROI, colored according to (D) the original/local, (E) median, and (F) mean helix angle.

**Figure 2:**
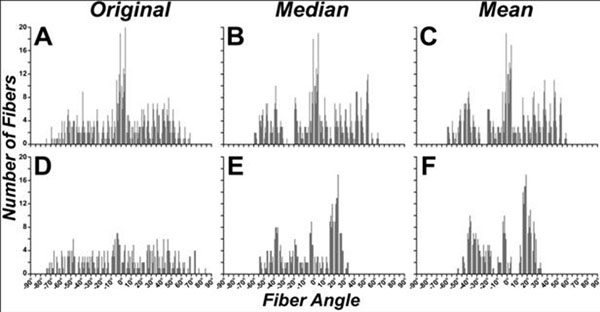
(A) Histogram of fiber helix angles sampled at original/local points along fiber trajectories within the region of interest (ROI). (B, C) Histogram of (B) median and (C) mean fiber helix angles in the ROI. (D) Helix angle histogram of fibers passing through the ROI, (E) histogram of median helix angles and (F) mean helix angles. Fiber lengths were limited to the ROI in (A-C) and to half the mid-ventricular circumference in (D-F).

The impact of the helix angle classification scheme was dependent on fiber length and the noise in the dataset. Helix angles were similar with all approaches when fiber length was limited to the ROI (Figure 1A-C). Significant differences, however, were seen when fiber length was equal to half the circumference of the left ventricle (πR) (Figure 1D-F). The median classification excluded noisy/outlier voxels and produced a helix angle pattern with greater angular resolution than the mean classification scheme, which is affected by outlier voxels. The greater angular resolution produced by the median approach can be seen in the epicardial (red) fibers in Figure 1D-F and the histograms in Figure 2D-F. Far greater differences between the median and mean approaches are seen with noisier datasets. Fiber classification by local/original helix angle revealed that the helix angle along myofibers tended to be lower (more circumferential) at the apex and base than at the midventricular level (Figure 1D).

## Conclusions

The helix angle along a given myofiber is not constant and is highest at the midventricular level. The classification of myofibers based on their median helix angles strikes the optimal balance between clarity and preserved angular resolution.

